# ﻿Resolving a nearly 95-year-old enigma: Transfer of the little-known Japanese moss *Arctoaschistioides* to *Kiaeriafalcata* (Rhabdoweisiaceae, Bryophyta)

**DOI:** 10.3897/phytokeys.254.141498

**Published:** 2025-03-28

**Authors:** Wen-Zhuan Huang, Jia-Yi Zheng, Xin-Rui Xia, Xin-Yin Ma, Tian-Xiong Zheng, Yu-Huan Wu

**Affiliations:** 1 College of Life and Environmental Sciences, Hangzhou Normal University, Hangzhou 311121, China Hangzhou Normal University Hangzhou China; 2 Hattori Botanical Laboratory, Obi 6-1-26, Nichinan, Miyazaki 889-2535, Japan Hattori Botanical Laboratory Miyazaki Japan

**Keywords:** Bryophyta, *Dicranumschistioides*, doubtful species, new synonym, taxonomy

## Abstract

Taxonomic uncertainties regarding rare species often impede effective biodiversity conservation. One such taxonomic uncertainty is the 95-year-old mystery surrounding *Arctoaschistioides* (Broth. ex Ihsiba) Ihsiba. Since its initial publication in 1929, this species has not been subjected to any further discoveries and is, thus, classified as “doubtful taxa” or “insufficiently known taxa” to date. Assessing the taxonomic status of this species is essential for determining whether a conservation strategy should be implemented. In this study, we examined the holotype of *A.schistioides* and treated this species as a new synonym of *Kiaeriafalcata* (Hedw.) I.Hagen, a widely distributed species in the Northern Hemisphere, by providing detailed description, illustration and taxonomic notes. Our findings not only resolve this long-standing mystery, but also enhance our understanding of Japanese mosses and the global distribution of bryophytes.

## ﻿Introduction

Japan is renowned for its extensive diversity of mosses and is regarded as one of the global centres of moss diversity (Geffert et al. 2013). In the most recent checklist, Suzuki (2016) documented 1,270 species across 342 genera within the Japanese moss flora; however, 149 species were classified as “doubtful taxa” in this checklist. Resolving the uncertainties surrounding these questionable species is crucial for enhancing our understanding of Japanese moss diversity and the global distribution of bryophytes.

**Figure 1.**
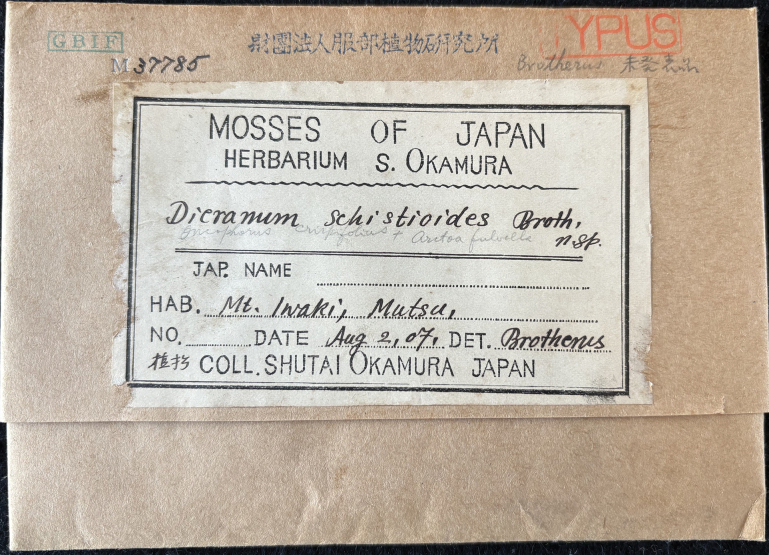
Specimen label of the holotype of *Arctoaschistioides* (Broth. ex Ihsiba) Ihsiba (*S. Okamura s.n.* [NICH 37785]).

*Arctoaschistioides* (Broth ex Ihsiba) Ihsiba is one such enigmatic species, possessing a noteworthy taxonomic history. In 1907, S. Okamura collected an interesting specimen from Mt. Iwaki, Japan (Fig. 1), which was initially identified by V.F. Brotherus as a new species and later compiled and published by Ihsiba (1929) as “*Dicranumschistioides* Broth. ex Ihsiba”. Subsequently, Ihsiba (1932) transferred this name to *Aratoaschistioides*. Sakurai (1954) provided a comprehensive catalogue on Japanese mosses, but did not recognise the species. Iwatsuki and Noguchi (1973) listed all genera and species of mosses in Japan and considered *A.schistioides* a well-established taxon, which was followed by Sekine (1982). Since then, the taxonomic status of *A.schistioides* started to be questioned. Crosby et al. (1999) classified this name as “insufficiently known” in “A Checklist of the Mosses”. In subsequent versions of the Japanese moss checklist (e.g. Iwatsuki (2004, 2011); Suzuki (2016)), the distribution and record of *A.schistioides* in Japan was considered doubtful. In fact, this species has not been subjected to any additional discoveries or descriptions in the 95 years since its publication (Ihsiba 1929), resulting in very limited knowledge about it. To date, the status of *A.schistioides* remains an enigmatic issue requiring further resolution.

Accurate species identification is crucial for biodiversity conservation, particularly amongst rare taxa that are taxonomically uncertain due to insufficient study (Ding et al. 2018; Li et al. 2023). Erroneous classification and misidentification may overlook endangered species that warrant protection (Gibson et al. 2019). Conversely, management actions stemming from incorrect species identification can waste resources and funding (Solow et al. 2011). Therefore, further assessment is necessary to clarify the taxonomic status of *Arctoaschistioides*.

## ﻿Material and methods

Specimen of *Arctoaschistioides* (≡ *Dicranumschistioides*), probably the holotype (*S. Okamura s.n.*; NICH 37785), was borrowed from NICH and morphologically examined. Notes on the nomenclatural status and collection site of this specimen were provided below.

The specimen was examined by using a stereomicroscope (Leica EZ4; Leica, Wetzlar, Germany) and a compound microscope (Leica DM6 B; Leica, Wetzlar, Germany). Microscopic pictures were captured using a digital camera (Leica DFC450 C; Leica, Wetzlar, Germany) attached to the compound microscope. The plant pictures were taken using a stereomicroscope (Keyence VHX-6000; Keyence, Osaka, Japan).

## ﻿Results

Based on morphological observations, *Arctoaschistioides* exhibits no morphological differences from *Kiaeriafalcata* (Hedw.) I.Hagen. Therefore, we treat *A.schistioides* as a new synonym of *K.falcata*.

### ﻿Taxonomic treatment

#### ﻿ Kiaeria falcata

Taxon classificationPlantaePlatycopidaRhabdoweisiaceae

(Hedw.) I.Hagen, Kongel. Norske Vidensk. Selsk. Skr. (Trondheim) 1914(1): 112. 1915.

EF252353-5145-5B32-AB6E-62F66B903484

= Arctoaschistioides (Broth. ex Ihsiba) Ihsiba, Classif. Mosses Japan: 130. 1932. syn. nov.
≡ Dicranumschistioides Broth. ex Ihsiba, Cat. Mosses Japan: 43. 1929.

##### Type.

**Figure 2.**
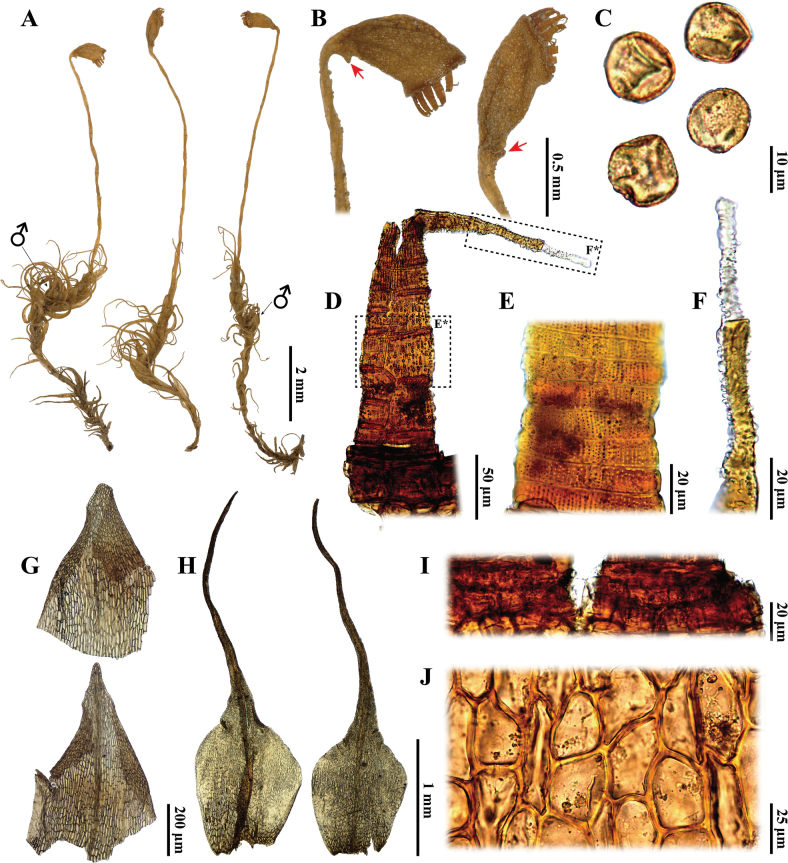
*Arctoaschistioides* (Broth. ex Ihsiba) Ihsiba **A** plants **B** capsules, arrows shows strumose **C** spores **D, E, F** peristome teeth **G** perigonial leaves **H** perichaetial leaves **I** annulus **J** exothecial cells. All from the holotype (*S. Okamura s.n.* [NICH 37785]).

**Figure 3.**
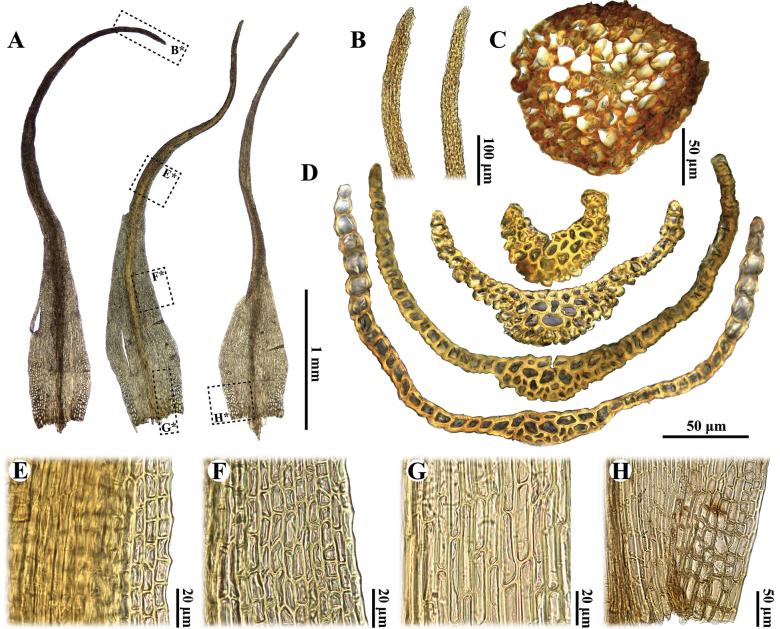
*Arctoaschistioides* (Broth. ex Ihsiba) Ihsiba **A** leaves **B** apex **C** cross section of stem **D** cross sections of leaf **E** upper laminal cells of leaf **F** middle laminal cells of leaf **G** basal juxtacostal cells **H** alar cells. All from the holotype (*S. Okamura s.n.* [NICH 37785]).

Japan • Aomori Prefecture, Hiromae City, Mt. Iwaki, 2 Aug 1907, *S. Okamura s.n.* (***holotype***: NICH 37785!), (Figs 2, 3).

##### Description.

Plants small, in loose tufts. Stems 5–8 mm, simple, cross-section of stem rounded to oval, diameter 0.11–0.15 mm, central strand present. Leaves homomallous, falcate-secund when dry, erect-spreading when moist. Leaves lanceolate at base, gradually tapering into a channelled acumen; costa excurrent as an awn, mamillose dorsally, in transverse section with differentiated guide cells, with dorsal and ventral epidermis and few substereids on dorsal side of guide cells or poorly differentiated; margins plane or slightly incurved distally, entire or crenulate in distal part of acumen; lamina unistratose, occasionally partially 2-stratose in distal portion, margins 1-stratose; distal and median laminal cells short rectangular to subquadrate, with moderately thickened walls, (6–)8–15(–19) × 4–8 µm; basal juxtacostal cells elongate-rectangular, moderately thick-walled, non-porose, 30–50 × 5–9 µm; alar cells gradually enlarged, not sharply differentiated, unistratose, scarcely inflated, composed of short-rectangular to quadrate inflated cells, non-porose, 24–45 × 14–20 µm.

Autoecious. Perigonia terminal closely located below the perichaetia. Perigonial leaves small, ovate-lanceolate to triangular, 0.65–0.75 × 0.45–0.55 mm, costa present or absent; Perichaetial leaves with sheathing base, abruptly into a channelled acumen. ca. 3.2 mm long. Sporophyte single in perichaetium. Seta straight, 5.5–7.5 mm long, yellowish-brown. Capsules obovate, curved and strumose, smooth when dry; Exothecial cells irregular, short rectangle, thick walled; Calyptra not seen; Operculum not seen; Annulus persistent, one row of small cells; Peristome teeth to 0.35 mm long, orange-brownish below, whitish in distal portion, divided into two prongs to the middle, vertically pitted-striolate below, papillose above. Spores 14–17 µm.

##### Notes.

The nomenclatural status of the cited specimen (*S. Okamura s.n.*; NICH 37785) should be stated first. According to the protologue of *Dicranumschistioides* (Ihsiba 1929), the type specimen of this species was collected from “津軽富士” (Tsugarufuji; in English), which is another name for Mt. Iwaki (岩木山; in Japanese) in Aomori Prefecture, Japan (Tokuhisa 1978). During this study, we extensively searched the bryological collection of NICH and located only one specimen of *D.schistioides*, namely “*S. Okamura s.n.*” (NICH 37785), which was detected by V. F. Brotherus and collected by S. Okamura from “Mt. Iwaki, Mutsu” (Fig. 1). Since Aomori Prefecture was administratively part of “Mutsu” (Sanseidohenshuusho 1975), we thus deemed that this specimen shares the same collection site as recorded in the protologue of *D.schistioides* (Ihsiba 1929). Furthermore, as the original collection of S. Okamura was supposedly deposited in NICH (Vitt et al. 1985), it can be thus inferred that the present specimen is the holotype of *D.schistioides* (Art. 9.1; Turland et al. 2018). As Mt. Iwaki (Fig. 1) is now included in Hiromae City, Aomori Prefecture, we provided a corrected type citation above.

In addition, nomenclature of *Dicranumschistioides* also needs a brief discussion here. This species is nomenclaturally valid although it was only described in Japanese at the time of publication (Art. 39.1; Turland et al. 2018). Later, it was transferred to the genus *Arctoa* without providing a basionym or replaced synonym (Ihsiba 1932). However, the taxonomic authority of its basionym “(Broth.)” and Japanese name “たかねかもじごけ” were clearly given, which should be regarded as an indirect reference (Arts. 38. 14 & 41.3; Turland et al. 2018), giving *A.schistioides* a valid taxonomic status.

*Arctoaschistioides* is distinguished by the following characteristics: (1) a strumose capsule (Fig. 2A, B), (2) a smooth capsule when dry (Fig. 2B); (3) the presence of a central strand (Fig. 3C), (4) gradually enlarged alar cells (Fig. 3A, H), (5) a nearly homogeneous costa structure without stereids (Fig. 3D), (6) mamillose leaf subula (Fig. 3B, D), (7) distal laminal cells that are subquadrate to short rectangular (Fig. 3E), (8) elongate-rectangular basal juxtacostal cells (Fig. 3G), (9) a persistent annulus comprised of small cells (Fig. 2I), (10) perigonia located just below the perichaetia (Fig. 2A) and (11) irregular, short rectangle, thick-walled exothecial cells (Fig. 2J). These characteristics imply that *A.schistioides* actually belongs to *Kiaeriafalcata* due to the lack of distinct morphological differences between the two species (Newmaster 2007a; Brugués and Ruiz 2012; Lüth 2019).

Morphologically, *Arctoaschistioides* may be confused with *A.fulvella* (Dicks.) Bruch & Schimp. due to their similar plant morphology and leaf shape (Noguchi 1987; Newmaster 2007a, 2007b; Lüth 2019). However, the alar cells of *A.schistioides* are gradually enlarged and not sharply differentiated (Fig. 3A, H), whereas those of *A.fulvella* are clearly delimited and well differentiated (Noguchi 1987; Gao et al. 1999; Ochyra and Buck 2003; Newmaster 2007b; Lüth 2019). Additionally, these two species can be distinguished by their capsule morphology: *A.schistioides* exhibits smooth capsules when dry, characterised by a distinct strumose (Fig. 2A, B). In contrast, *A.fulvella* possesses distinctly ribbed capsules when dry and lacks a strumose structure (Noguchi 1987; Ochyra and Buck 2003; Newmaster 2007b; Lüth 2019). Notably, both species share peristome teeth that are divided into two prongs at the mid-point (Fig. 2D; Noguchi (1987); Newmaster (2007b)); however, this division is not always conspicuous in *A.fulvella*, as the teeth occasionally appear undivided and perforated near the middle (Gao et al. 1999; Ochyra and Buck 2003; Lüth 2019).

*Arctoaschistioides* is easily confused with *A.blyttii* (Bruch & Schimp.) Loeske. However, the leaves of *A.schistioides* are homomallous and falcate-secund when dry (Fig. 2A), while those of *A.blyttii* are erect-spreading and flexuose (Newmaster 2007a; Brugués and Ruiz 2012). Additionally, the perigonia of *A.schistioides* are situated just below the perichaetia (Fig. 2A), whereas those of *A.blyttii* are terminal on a separate branch or positioned far below the perichaetia (Newmaster 2007a; Brugués and Ruiz 2012). Another distinguishing feature is that the exothecial cells of *A.schistioides* are irregular, short rectangular and thick-walled (Fig. 2J), while those of *A.blyttii* are rectangular and thin-walled (Brugués and Ruiz 2012). Furthermore, the annulus of *A.schistioides* is persistent and consists of a single row of small cells (Fig. 2I), whereas the annulus of *A.blyttii* is deciduous and composed of three rows of large cells (Brugués and Ruiz 2012, 2015).

*Arctoaschistioides* is also morphologically similar to *A.starkei* (F. Weber & D. Mohr) Loeske and *A.glacialis* (Berggr.) Fedosov, Jan Kučera & M. Stech. However, the upper laminal cells of the latter two species are long and rectangular and their capsules are ribbed or grooved when dry (Newmaster 2007a). In contrast, the upper-middle cells of *A.schistioides* are short rectangular to subquadrate (Fig. 3E) and its capsules are smooth when dry (Fig. 2A, B). Additionally, Kiaeriafalcatavar.serratifolia Sakurai, a taxon endemic to Japan, can only be distinguished from *A.schistioides* by its serrate leaf margins (Sakurai 1952), whereas the latter species are smooth or crenulate leaf margins in the distal part of the acumen (Fig. 3A, B).

*Dicranumhakkodense* Cardot, an intriguing species that shares the Japanese name “タカネカモジゴケ” with *Arctoaschistioides*, but can be distinguished from the latter species by several characteristics. The leaf tips of *D.hakkodense* are moderately fragile and the leaves are straight or only slightly falcate-secund when dry (Ignatova and Fedosov 2008; Huang et al. 2023, 2024). In contrast, the leaf tips of *A.schistioides* are robust, the leaves are homomallous and falcate-secund when dry (Fig. 2A). Furthermore, *D.hakkodense* exhibits clearly differentiated alar cells, a cross section of the costa that contains distinct stereids and a capsule that lacks strumose features (Ignatova and Fedosov 2008; Huang et al. 2023, 2024). Conversely, *A.schistioides* possesses alar cells that are not sharply differentiated (Fig. 3A, H), a cross section of the costa that lacks stereids (Fig. 3D) and a capsule that exhibits strumose features (Fig. 2A, B).

In conclusion, we propose *Arctoaschistioides* as a new synonym of *Kiaeriafalcata*.

## Supplementary Material

XML Treatment for Kiaeria falcata

## ﻿Additional information

### Author ORCIDs

Wen-Zhuan Huang https://orcid.org/0000-0001-5871-5699

Jia-Yi Zheng https://orcid.org/0009-0000-6222-8818

Xin-Rui Xia https://orcid.org/0009-0006-7708-7716

Xin-Yin Ma https://orcid.org/0000-0003-0876-4497

Tian-Xiong Zheng https://orcid.org/0000-0002-1963-2302

Yu-Huan Wu https://orcid.org/0000-0002-8139-2194

### Data availability

All of the data that support the findings of this study are available in the main text.
